# The Nursing Staff's Understandings of Spiritual Care in an Oncology Clinic in 2003 and 2023: A Mixed Method Longitudinal Study

**DOI:** 10.1111/jan.16738

**Published:** 2025-01-03

**Authors:** Mikael Lundmark

**Affiliations:** ^1^ Department of Historical, Philosophical and Religious Studies Umeå University Umeå Sweden

**Keywords:** case study, longitudinal study, nursing, oncology, religiosity, religiousness, spiritual care, spirituality

## Abstract

**Aim(s):**

To explore the understandings of spiritual care among nursing staff at an Swedish oncology clinic, with a special focus on changes over time.

**Design:**

Qualitative, longitudinal, descriptive design.

**Methods:**

A questionnaire‐based replication study conducted in 2003 (*N* = 68) and 2023 (*N* = 47), comparative and thematic analysis.

**Results:**

The thematic analysis generated four main themes in 2023: (i) Relate to the whole person—spiritual care as an approach, (ii) enable an atmosphere of humanity and security around the patient—spiritual care as a nursing intervention, (iii) enable the patient to live out their spirituality or practice their religion—spiritual care that focuses on practical aspects directly linked to practising a religion and (iv) awareness of the importance of one's own approach to spiritual care—spiritual care that focuses on the personal qualities of the caregiver. Compared with 2003, there are many similarities but also some differences: the importance of one's own approach is more emphasised in 2023, and there is an increased awareness that nursing interventions can be different depending on who the patient is and who the caregiver is; spiritual care is more clearly characterised by an effort to relate to the whole person and the understanding of what can/should be included in the framework of spiritual care is broader.

**Conclusion:**

The concept of ‘spiritual care’ can change over time and can depend on societal changes.

**Implications for the Profession and/or Patient Care:**

When studying understandings of the concept of ‘spiritual care’ in a nursing context or evaluating studies on the topic, the stability of the concept of ‘spiritual care’ over time should be accounted for.

**Reporting Method:**

When applicable: SRQR.

**Patient or Public Contribution:**

None.

**Summary**

- Impact
  ○ The understandings of spiritual care in one Swedish oncology clinic and whether such understandings change over time.
  ○ Many similarities but also some differences between 2003 and 2023.
  ○ This research will guide further studies on nursing staff's understandings of ‘spiritual care’.

## 1 Introduction

Awareness of patients' spiritual needs has increased substantially since the turn of the 21st century (O'Brien 2022). The connection between spirituality (as it relates to religion) and health has been well established in numerous studies (Koenig, VanderVeele, and Peteet 2024). The implications of this awareness have been studied and discussed in various nursing contexts, including critical care (García Torrejon et al. 2023), mental health nursing (Elliott et al. 2019), general practice nursing (Schreiber, Verrall, and Whitehead 2022), nursing home care (Santos et al. 2021) and palliative care (de Brito Sena et al. 2021). Recurring themes and topics in research on spirituality and healthcare include determinants behind spiritual care practices (Mascio et al. 2022), competencies needed for spiritual care (Espinel et al. 2022), challenges associated with spiritual care (Balboni et al. 2014) and how the concept of ‘spirituality’ (de Brito Sena et al. 2021) and ‘spiritual care’ (Hvidt et al. 2020; Lundberg et al. 2024) can be understood.

Several methodological and conceptual problems associated with research on spirituality and health have been noted (Reinert and Koenig 2013; Chagas et al. 2023). Regarding research on how the concepts ‘spirituality’ and ‘spiritual care’ are understood in healthcare contexts, concerns have been raised regarding the impact cultural differences may have on these concepts (Torskenæs et al. 2015). Notwithstanding this, a question that has not been previously addressed in the literature, but which is essential when comparing research on understandings of spiritual care conducted at different times, is whether understandings of the concept of ‘spiritual care’ remain stable over time or whether they are sensitive to societal changes, for example.

To investigate this question, a study was conducted in 2023, replicating a study that had been made 20 years earlier on nursing staff's attitudes to, understandings of and perceived difficulties associated with spiritual care, in a holistic nursing context at a Swedish oncology clinic (Lundmark 2003, 2005, 2006). In this context, *holistic care* refers to a response to an individual's physical, psychological, social and spiritual needs (Lundmark 2024).

Since parts of the 2003 study was concerned with examining the staff members' attitudes towards spiritual care and perceived difficulties with providing spiritual care, it was deemed essential to ensure that all the respondents related to the same understanding of the concept of ‘spiritual care’, thereby ensuring that a ‘meeting of minds’ took place. To this purpose, a tentative working definition of the concept of ‘spiritual care’ was used in the 2003 study and reused verbatim in the 2023 study. The definition was informed by the results of Strang, Strang, and Ternestedt's (2002) study of how Swedish nursing staff at seven different healthcare units characterise their patients' spiritual needs. The definition of the concept was formulated as

making possible/facilitating for the patient, with the help of suitable nursing interventions, to express and discuss existential questions and to practise his/her spirituality (which may be done through the practising of a specific religion but also through activities which do not need to be of religious nature). (Lundmark 2006, 868)

It was also of interest to find out whether the nursing staff agreed on this definition or whether they understood ‘spiritual care’ in different ways. Consequently, a part of the 2003 study focused on examining the nursing staff's understanding of the concept of ‘spiritual care’.

### 1.1 Societal Changes Related to the ‘Spiritual Climate’ in Sweden Between 2003 and 2023

During the 20 years that have passed since the 2003 study, the spiritual climate in Sweden has changed. When we consult the statistics for Sweden in Wave 4 in the World Values Survey (WVS) (covering the years 1999–2003) and compare its position in Wave 7 (covering the years 2017–2022), we note that in both surveys that Sweden is one of the countries with the highest factor scores for ‘secular values’. Sweden also scored the highest factor scores for ‘self‐expression’. Between Wave 4 and Wave 7, Sweden's factor scores for ‘self‐expression values’ increased from 2.8080 to 3.1133. ‘Self‐expression values’ reflect a humanistic and emancipative ethos where human autonomy and choice are emphasised (Inglehart et al. 2014). Furthermore, as Thurfjell (2015) and Davidsson Bremborg (2008) have argued, ‘self‐expression values’ are relevant to understanding certain aspects of Swedish spirituality. An example of this is the trend in the Swedish society towards an ever‐increasing individualisation and privatisation of spirituality that includes an increasing diversity of how a person's spirituality might be expressed (Thurfjell 2015). In an international perspective, for example, comparing with the United States during Wave 4, the population of the United States seemed to be more religious than the population of Sweden, for example, in terms of ‘belief in God’. In the WVS 4 (1999–2004), 94.2% of the population of the United States was reported to believe in God. In Sweden, on the other hand, the corresponding figure was 46.6% (Inglehart et al. 2014).

The proportion of the Swedish population that reported a belief in God decreased between Wave 4 and Wave 7 from 46.6% to 34.4%, respectively. Similarly, the proportion of the population that reported that they do not believe in God increased from 40.6% to 60.8% during the same period. Similar trends can be seen regarding belief in a life after death and participation in religious meetings (Inglehart et al. 2014; Haerpfer et al. 2022).

The results of the parts of the replication study presented here are discussed with particular reference to the previous examples given regarding societal changes related to the Swedish spiritual climate. The parts of the study dealing with the nursing staff's perceived difficulties associated with spiritual care, their attitudes towards spiritual care and what influences these attitudes will be reported elsewhere (Lundmark Forthcoming a, Forthcoming b).

## 2 The Study

### 2.1 Aims and Research Questions

The present article is based on some of the results of the 2023 replication study of Lundmark's study conducted in 2003. It has two aims:

The first one is to replicate the 2003 study with the following research question:

RQ1 How do the nursing staff at a Swedish oncology clinic describe the concept of ‘spiritual care’?

The second aim is to perform a comparative analysis of the results from the 2003 study and the 2023 study to identify similarities and differences between the results. This work is a response to the following research question:

RQ2 Has the nursing staff's understanding of the concept of ‘spiritual care’ changed between 2003 and 2023 in a Swedish holistic nursing context (represented by an oncology clinic)? If their understanding has changed, then in what ways?

The answers to these research questions, in conjunction with the examples provided in the introduction of this article regarding societal changes related to spirituality (i.e., a decline in religiousness and an increase in self‐expression values), inform the following discussion of the concept of ‘spiritual care’, including whether it has remained stable over time and is sensitive to societal changes, as well as the relevance of the results across different cultures.

## 3 Methods

### 3.1 Procedure

To gather as comparable data as possible, the choice was to do an as exact replication study as possible on the same clinic as where the original study was conducted. Therefore, the research procedure used in the 2023 study followed the procedure of the 2003 study as closely as possible. However, because the oncology clinic in question has undergone several structural changes during the past 20 years and because of changes in the Swedish society during the same period, the 2023 study's design was not exactly the same as that of the 2003 study. At the time of the 2003 study, the clinic consisted of three inpatient wards and one small outpatient ward. In 2023, the three inpatient wards were merged into one large inpatient ward. By 2023, the outpatient ward was considerably larger than what it was 20 years earlier. In 2023, there were fewer nursing staff members at the clinic, but a larger proportion of the 2023 nursing staff were registered nurses, and considerably more nursing staff worked on the outpatient ward. The change in the Swedish society that impacted the design of the 2023 study included the fact that the number of Muslims in Sweden was higher in 2023 than that in 2003 (Swedish Agency for Support to Faith Communities 2024). This demographic change is reflected in how organised religiousness and non‐organised religiousness were assessed in the questionnaire, where the words *mosque* and *the Koran* were included with the words *church* and *the Bible* as examples of religious institutions and religious texts, respectively.

A paper questionnaire and a short information letter that described the intended study were handed out to all the registered nurses and nursing auxiliaries in the oncology clinic's inpatient and outpatient wards (for the 2023 study, *n* = 82; for the 2003 study, *n* = 93) who were on duty during the last week of January 2003/2023 and the first week of February 2003/2023. Oral information about the study was given to the staff members as well. Participation in the study was voluntary and anonymous. The questionnaires were submitted by the participating nursing staff in sealed letterboxes that were located in the wards.

### 3.2 The Questionnaire

The 2003 study's research questions were operationalised in the form of a questionnaire that included the open‐ended question, *How would you describe the term spiritual care?* (cf. Strang, Strang, and Ternestedt 2002; Wright 2002). The questionnaire also included an open‐ended question designed to assess perceived difficulties to providing spiritual care (Lundmark 2005, Forthcoming b). In addition to this, the questionnaire also included 17 closed questions designed to assess data about attitudes to spiritual care and determinants of these attitudes, including numbers of years working in health care, belief in God and life after death, and organised religiousness and non‐organised religiousness (Lundmark 2006, Forthcoming a). Some of the responses to the 17 closed questions are referred to in this article as background data (see Tables 1, 2, 3, 4) and are inspired by Koenig et al. (2001, 496–500), except the question, *Do you believe in life after death?*, which was taken from the Fetzer Institute/National Institute on Aging Working Group (1999). Organised religiousness (Table 3) and non‐organised religiousness (Table 4) were assessed with one question each. Religious belief was assessed with two questions (Table 2). These questions were designed prior to the 2000s, before the distinction between religiousness and spirituality was as distinct as it has become today (see, e.g., Koenig, VanderVeele, and Peteet 2024) and mirrors what Schnell (2012) label as ‘spirituality with religion’ in contrast to ‘spirituality without religion’.

**TABLE 1:** Number of years working in health care.

Question	Distribution of answers						
	0–2 years	3–8 years	9–14 years	15–20 years	More than 20 years	Missing answers	*n*
How many years have you worked in health care?	6% (7%)	11% (19%)	17% (12%)	17% (22%)	49% (40%)	0 (0)	47 (68)

*Note:* The figures from 2023 are given without parenthesis and the figures from 2003 are given in parenthesis.

**TABLE 2:** The respondents' religious beliefs measured as belief in God and in life after death.

Questions	Distribution of answers				
	Yes	No	Do not know	Missing answers	*n*
Do you believe in God?	40% (53%)	47% (24%)	13% (22%)	0 (1)	47 (67)
Do you believe in life after death?	34% (56%)	30% (22%)	36% (22%)	0 (0)	47 (68)

*Note:* The figures from 2023 are given without parenthesis and the figures from 2003 are given in parenthesis.

A methodological dilemma arose during the construction of the questionnaire, namely, whether the respondents should be given a tentative definition of the concept of ‘spiritual care’ or whether they should associate freely around this concept. An advantage of providing a definition would have allowed all the respondents to interpret the questions about spiritual care similarly. A disadvantage to such an approach is that it would have forced the respondents into one interpretation of ‘spiritual care’ out of several possible interpretations, thus rendering their personal view more difficult to identify. Due to practical reasons associated with the statistical analysis of the data (Lundmark [Link], 2006), it was decided to include the tentative definition, earlier referred to, of the concept of ‘spiritual care’ in the questionnaire. The questionnaire was structured so that the respondents were given the definition of spiritual care in the beginning of the questionnaire and were asked to consider this definition when answering the 17 closed questions. The open‐ended questions (including the question about their individual understanding of spiritual care) was placed at the end of the questionnaire.

The scientific community validated the questionnaire during the 2003 study through discussions with experienced researchers in nursing science, statistics and the social science of religion (cf. Kvale 1997). The questionnaire was tested in a pilot study in which six caregivers were asked to answer the questionnaire and give comments regarding the questionnaire's disposition. The responses garnered during the pilot study informed adjustments to the questionnaire to increase its clarity. The whole questionnaire (in Swedish) can be found in Lundmark (2003).

### 3.3 Analysis

The questionnaire responses that are presented in this article were analysed using a method of ‘content analysis’ as described by Downe‐Wamboldt (1992). Each answer to the open‐ended questions was compiled and printed out. These texts were read through to obtain a general feeling about what the answers addressed. The answers were reread and divided into meaningful units that were based on parts of the sentences, whole sentences or several sentences taken together. These substantive meaning units were then condensed and grouped into sub‐themes (In the 2003 study, these units were first grouped into sub‐sub‐themes.) and then, in turn, into themes. The answers were then read again and compared with the thematisations that had been made to verify that the procedure covered every aspect of each answer (Downe‐Wamboldt 1992; Lundmark 2005). This procedure was performed in the 2003 and 2023 studies by the same researcher, who possessed profound knowledge about the topic. This research approach is in accordance with Tuval‐Mashiach et al.'s recommendations when a research material is coded by one researcher only (Lieblich, Tuval‐Mashiach, and Zilber 1998, 133).

The Statistical Package for the Social Sciences SPSS 29.0.1.0 (2003: SPSS 11.00) generated descriptive statistics, summarising how the respondents' answers were distributed. The Fisher's exact test was also used for the statistics presented in this article.

### 3.4 Ethical Considerations

The study was approved by an ethical committee (Etikprövningsmyndigheten, Dnr 2022‐05805‐01), and approval from the head of the clinic and operation managers for the wards was secured.

## 4 Results

### 4.1 Response Rate

The 2003 study's response rate was 73% (of a total of 93 staff members). However, the response rate varied between the four oncology wards that participated in the study (the outpatient ward showed a response rate of 80% and the three inpatient wards showed a response rate of 94%, 60% and 59%). In all, 46 (68%) of the participating staff who handed in a questionnaire answered the questions regarding their understanding of ‘spiritual care’. In the 2023 study, the response rate was 57% (of in total 82 staff members) but varied somewhat between the wards. For the inpatient ward, the response rate was 45% (of a total of 49 staff members), whereas the response rate for the outpatient ward was 76% (of a total of 33 staff members). A total of 31 (66%) of the participating staff who handed in the questionnaire in the 2023 study answered the questions about their understanding of ‘spiritual care’.

### 4.2 Subjects

Among the 47 respondents in the 2023 study (the 2003 study had 68 respondents), 73% were registered nurses (in 2003: 57%) and 25% were nursing auxiliaries (in 2003: 40%). Table 1 shows the distribution among the respondents regarding how many years they had worked in healthcare. The distribution was quite similar in 2023 to what it was in 2003. According to one of the operation managers, approximately one‐third of the staff working in 2023 had worked in the clinic for at least 20 years and thus could have participated also in the 2003 study.

Two questions explored the distribution of religious belief: *Do you believe in God?* (‘yes’/‘no’/‘do not know’) and *Do you believe in life after death?* (‘yes’/‘no’/‘do not know’). The distribution of these answers is presented in Table 2. The number of staff who reported that they believed in God, as well as believed in a life after death, decreased between 2003 and 2023 by a statistically significant amount concerning the former (*p* = 0.036) but not concerning the later.

The distribution of the responses concerning a belief in life after death shows similarities to the distribution of responses concerning a belief in God, including the direction of the changes between 2003 and 2023, but these changes are not statistically significant.

The distribution of the answers to the questions concerning organised religiousness and non‐organised religiousness can be seen in Tables 3 and 4, respectively. It is evident that in 2023, the staff were less engaged in religious activities related to organised religiousness compared with 2003. However, the difference in reported engagement is not statistically significant. The differences concerning non‐organised religiousness between 2003 and 2023 are even smaller.

**TABLE 3:** Organised religiousness of the respondents.

Question	Distribution of answers						
	Never	At most once a year	A few times per year	Around once or twice a month	About every week	Missing answers	*n*
How often do you attend religious meetings (for example in a church or mosque)?	30% (22%)	45% (37%)	11% (27%)	6% (6%)	8% (7%)	0 (1)	47 (67)

*Note:* In the 2003 study, the question was instead formulated like this: ‘How often do you go to church or attend religious meetings?’ The figures from 2023 are given without parenthesis and the figures from 2003 are given in parenthesis.

**TABLE 4:** Non‐organised religiousness of the respondents.

Question	Distribution of answers						
	Never	A few times per year	A few times a month	Some times a week	Every day	Missing answers	*n*
How often do you spend time with activities like praying, meditation or reading religious literature (e.g., the Bible or the Koran)?	53% (47%)	19% (18%)	9% (6%)	13% (19%)	6% (10%)	0 (1)	47 (67)

*Note:* In the 2003 study, the question was instead formulated like this: ‘How often do you spend time with activities like praying, meditation or reading the Bible?’ The figures from 2023 are given without parenthesis and the figures from 2003 are given in parenthesis.

A comparison of the results of the 2023 and 2003 studies regarding opinions and attitudes towards spiritual care (accounted for in detail in Lundmark Forthcoming a) shows similar results concerning the respondents' opinions about holistic care where they, to a high degree, considered it important in nursing. The respondents also reported that in both 2003 and 2023, holistic care should include spirituality. However, fewer staff members thought that holistic care was provided in their respective wards in 2023 compared to that in 2003. The respondents' opinions regarding whether nursing staff should provide spiritual care increased from approximately two‐thirds of the staff in 2003 to three‐fourths in 2023. Estimations of whether the nursing staff in their ward provided spiritual care were quite similar between 2003 and 2023, where approximately one‐third of the staff reported that they did not know whether it was provided or not. However, there was a slight increase between 2003 and 2023 in the proportion of staff members who reported that they thought that spiritual care was provided in their respective wards.

Regarding the 2023 nursing staff's self‐estimation of how often they provided spiritual care, 10% of the staff reported that they provided spiritual care often or even daily, an increase from 3% in 2003. The proportion of staff members who reported that they never or hardly ever provided spiritual care decreased from approximately 54% in 2003 to approximately 40% in 2023. The respondents' self‐estimated ability to provide spiritual care slightly increased in 2023 compared to that in 2003.

Education in spiritual care was considered to be quite important by the nursing staff in both 2023 and 2003, with a slight increase being recorded in 2023. In contrast, a smaller proportion of the 2023 nursing staff thought that they had received education in spiritual care, either during their formal education or through their work.

Regarding sentiments of being at ease/not at ease if/when providing spiritual care, some small changes between 2003 and 2023 were observed. However, note that none of the above mentioned differences were statistically significant (Lundmark Forthcoming a).

### 4.3 Summary of the Nursing Staff's Descriptions of the Concept of ‘Spiritual Care’ in 2003

The thematic content analysis performed in the 2003 study generated three themes. These themes and associated sub‐themes can be found in Table 5. The following provides a summary discussion of these themes.

**TABLE 5:** Themes, sub‐themes and sub‐sub‐themes identified during the thematic content analysis of the answers to the question: How would you describe the concept of ‘spiritual care’? in the 2003 study.

Themes	Sub‐themes	Sub‐sub‐themes
Create space for spirituality	Enable patients to talk about existential issues with the nursing staff	As a healthcare professional, be open to the unfamiliar
		Dare to ask about/raise spiritual issues
	Enable patients to practice their religion during their stay in hospital	Provide information about the availability of a priest and psychologist
		Create opportunities for privacy
		Convey contacts
		Enable devotion at the clinic
		Enable singing at the clinic
		Enable reading at the clinic
	See the whole person	Be aware of the spiritual dimension of the person
	Include relatives in the provision of spiritual care	
Enable an atmosphere of humanity and security around the patients	Create an atmosphere that promotes patients' religious practices	Show respect for patients' spiritual needs
		In your role as a healthcare professional, do not influence patients with your views on religion
	Be present	Be a fellow human being
		Be available
		Meet the patient where he/she is
		Be open to the patient
		Be confident in front of the patient
		Take your time
		Have a keen ear
	Give your own experience	
	Give comfort	
	Give support	
	Show understanding	
	Do good to the patient	
	Have good thoughts about the patient	
	Show respect	Don't trivialise
		Don't push away
To involve the private spiritual dimension in the work		

*Note:* The table was translated from Swedish by the author and was originally published in Lundmark (2005, 34) and in unpublished form in Lundmark (2003, 32).

#### 4.3.1 Create Space for Spirituality

The theme ‘creating space for spirituality’ addressed how the respondents took the spiritual dimension into account and planned for it so that it was provided space on the ward. This theme was expressed in terms of how the staff members could enable their patients to raise existential questions with the staff. Formulations such as ‘conversations with the patients regarding existential questions’ and similar expressions were the most frequent wording reported in the questionnaire. Other ways of creating space for spirituality that were reported in the questionnaire included enabling patients to practice their religion during their stay at the clinic. This could be achieved by providing information about the priest and psychologist, creating opportunities for privacy or mediating contacts with the hospital church, for example.

‘Convey contacts’ was the second most frequent wording used in the nursing staff's responses. Creating space for spirituality could also take place by enabling various forms of devotional practice, for example.

Additional ways of creating space for spirituality included allowing the healthcare professional to view the patient as a whole person and involving relatives in the provision of spiritual care. ‘Seeing the whole person’ was interpreted as an expression that referred to a holistic view of patient care.

#### 4.3.2 Enable an Atmosphere of Humanity and Security Around the Patient

Enabling an atmosphere of humanity and security around the patient, among other things, entailed that healthcare staff be available for their patients and dedicated to their well‐being. In practical terms, this could include taking time to be with the patient, offering comfort and support, and showing empathy. Another way to facilitate an atmosphere of humanity and security was to create a mood in the ward that promoted the patients' religious practices.

Entertaining good thoughts about one's patients was also a way of enabling an atmosphere of humanity and safety for the patients, as well as being open and confident in front of the patients and sharing one's experiences with the patients. This approach included respect for one's patients' spiritual needs and an understanding that, as a healthcare professional, one must be careful not to influence the patient with one's own views on religion.

#### 4.3.3 Involve the Private Spiritual Dimension in the Nursing Staff's Work

This theme was referred to by a single statement by one respondent. Notwithstanding this, it was deemed necessary to present this theme because it addressed an issue that was very different from all the other themes that emerged in the survey material. One respondent claimed that one's private faith can constitute a concrete ‘work tool’ in nursing interventions. For example, a nurse might invoke the belief that it is both meaningful and part of good nursing to ask God for help as she helps her patients.

#### 4.3.4 The Proposed Definition of the Concept of ‘Spiritual Care’ Based on Strang, Strang, and Ternestedt (2002) and the Nursing Staff's Description of ‘Spiritual Care’ in 2003

As an alternative way of visualising the results of the content analysis, a new definition of the concept of ‘spiritual care’ in a nursing context was constructed. This definition was based on the initial definition of the concept included in the survey in the 2003 study and the content analysis that was made of the respondents' answers. This was done using the main themes (excluding the theme that was based only on one statement) to form a specification of ‘suitable nursing interventions’:

Spiritual care means, with suitable nursing interventions, enabling/facilitating the patient to raise and debate existential questions and live out their spirituality, which can take place through the practice of a specific religion but also through activities that do not have to be religious in nature. Suitable nursing interventions are characterised by efforts to create space for spirituality and an atmosphere of humanity and security around one's patients. (Lundmark 2005, 33–35, author's translation)

### 4.4 A Comparison of the Nursing Staff's Descriptions of ‘Spiritual Care’ in 2023 and 2003

In the 2023 study, the thematic content analysis generated four themes. These themes and sub‐themes are presented in Table 6. As each theme is presented, it is compared to the thematisation identified in the 2003 study. A summary of the results of the 2003 and 2023 studies is found in Table 7.

**TABLE 6:** The themes and sub‐themes based on a thematic analysis of the respondent's answers to the question: How would you describe the concept of ‘spiritual care’ in the 2023 study? Unlike the 2003 study, the results are presented as themes and sub‐themes only.

Themes	Sub‐themes
Relate to the whole person	See the whole person
	Show consideration for the patient and proceed from the patient's existential, psychological and spiritual needs
	Help balance the four dimensions (referring to Saunders' model with physical, psychological, social and spiritual or existential dimensions)
	Take the initiative in conversations about life issues and the like in conversations, musings and thoughts about existential dimensions (life, death, etc.)
	Support for anxiety and depression
	Everything that has to do with feelings
Enable an atmosphere of humanity and security around the patient	Give time and space
	Create security
	Be responsive and empathetic
	Be present
	Listen to the patient and try to understand her
Enable the patient to be able to live out their spirituality and practice their religion	Convey contacts
	Ensure that the patient is allowed to practice his religion
	Help the patient with practical religious practices (e.g., prayer, spiritual conversations) IF you, as a healthcare professional, feel that you CAN do it
Awareness of the importance of one's own approach to spiritual care	Be open and tolerant
	Not displaying one's own values
	Dare to talk about death and be realistic
	Show the patient that there is mental fortitude

**TABLE 7:** Summary table of the comparison between 2003 and 2023.

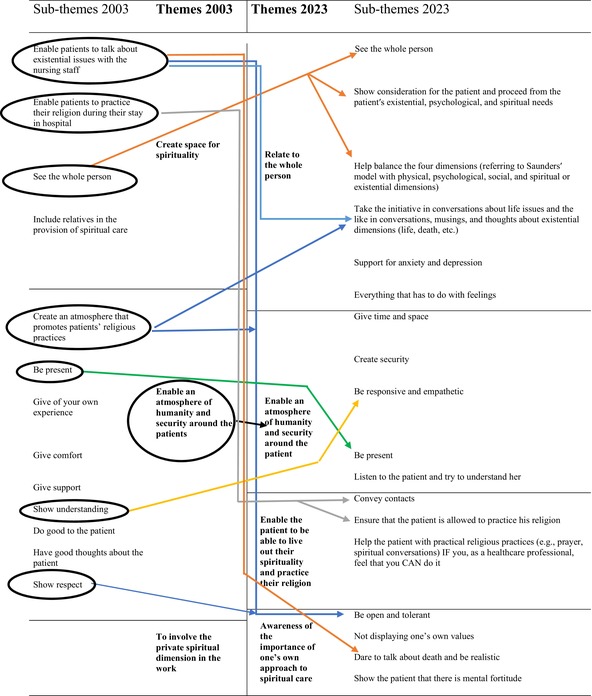

*Note:* Arrows indicate the similarities.

#### 4.4.1 Relate to the Whole Person—Spiritual Care as an Approach

This theme comprised approximately one‐third of the statements recorded in the questionnaire. It expresses a holistic care approach that includes showing consideration for the patient's existential, psychological and spiritual needs. This theme can also encompass providing support to the patient in case they succumb to anxiety and depression. Alternatively, it may involve the healthcare professionals taking the initiative to talk about life issues. It can also be understood in a more passive form, where the initiative to talk about life issues does not necessarily lie with the healthcare staff but perhaps with the patient instead, for example, by taking part in shared conversations, musings, and thoughts about life and death. This approach may also entail helping one's patients balance the four dimensions of Saunder's theory of ‘care thinking’, that is, the physical, psychological, social and spiritual or existential dimensions. In the statements included in the questionnaires that touched on this theme, a general understanding of ‘spiritual care’ was expressed as ‘everything that has to do with feelings’.

This theme has no direct counterpart in the thematisation of the questionnaire answers provided in 2003, but there is an inevitable overlap with the theme ‘create space for spirituality’ that emerged in the 2003 study. Related to this theme was the 2003 sub‐theme called ‘see the whole person’, where statements about the patient's spiritual dimension were found. In 2023, a different interpretation was made of this type of statement: they were primarily seen as the result of a holistic care approach, whereas in 2003, they were seen as a way of enabling spiritual care alongside measures of a concrete and practical nature. Furthermore, such statements were viewed as expressions of a positive approach to one's patients and their spirituality.

#### 4.4.2 Enabling an Atmosphere of Humanity and Security Around the Patient—Spiritual Care as a Nursing Intervention

This theme comprises almost half of the statements recorded in the questionnaire and focuses on the nursing staff's treatment of their patients. Related to this theme, we find a number of sub‐themes dealing with giving time and space to one's patients. Other related sub‐themes include creating security, showing understanding, and listening to and trying to understand one's patients; ‘trying to understand’ in this context is interpreted here as different from ‘showing understanding’. ‘Trying to understand’ refers to making an effort to understand one's patient, whether the patient is aware of this effort or not. On the other hand, ‘showing understanding’ refers to creating a feeling in the patient that the staff possesses an understanding of the patient's situation. Such an approach may also involve showing a humble and respectful attitude. A number of statements concerning the qualities of nursing caregivers were also included under this theme. Regarding this, reference was made to personal qualities that contribute to creating an atmosphere of humanity and safety around the patient, for example, being responsive, empathetic and present.

This theme was also identified in the 2003 study, but then it included sub‐themes which, in 2023, were judged to touch on an awareness of the importance of one's own approach to spiritual care, or to relate to the whole person.

#### 4.4.3 Enable the Patient to Live Out His Spirituality or Practice His Religion—Spiritual Care That Focuses on Practical Aspects Directly Linked to Practising a Religion

Somewhat more than one‐tenth of the statements in the questionnaire fall under this theme, which focuses on practical aspects that are directly linked to religious practice. Regarding this, several statements referred to mediating contacts, for example, to the hospital church and other institutions within the healthcare system and outside the system. This theme included helping the patient in their practical religious practices, such as engaging in prayer or spiritual conversations if the healthcare professional felt competent to do so. Alternatively, this theme may entail that the staff member made an effort to ensure that the patient was allowed to practice their religion. Note that the word *religion* was only mentioned in two of the respondents' answers.

This theme was not identified in the 2003 study. However, statements about mediating contacts were thematised in the 2003 study as part of creating space for spirituality.

#### 4.4.4 Awareness of the Importance of One's Own Approach to Spiritual Care—Spiritual Care That Focuses on the Personal Qualities of the Caregiver

This theme corresponds to approximately one‐tenth of the statements made in the questionnaire. To a large extent, this theme deals with the personal qualities of the caregiver, an observation that was manifested in an awareness of the importance of personal qualities for the implementation of spiritual care. Sub‐themes to this theme related to the qualities of the caregiver can also be associated with the theme ‘enable an atmosphere of humanity and security around the patient’, albeit with the critical difference that the present theme is focused on the nurse's awareness of the importance of their approach to whether and how spiritual care can be expressed. In this context, we find statements that referred to being open to and tolerant of one's patient's spirituality as a caregiver. This is related to the statement ‘Have an open mind, be tolerant’, for example. Furthermore, the caregiver is not expected to advocate for their own values when providing spiritual care, but this theme does address having the courage to talk about death and to confront the stark reality of the patient's circumstances. One statement recorded in the questionnaire raised the issue of showing the patient that healthcare professionals possess the strength of character to listen to challenging topics. The personal qualities that emerge in this theme can be summarised in terms of generosity (of spirit), courage and mental fortitude.

This theme was not found in the 2003 study, but similar statements in the 2003 study were thematised into the themes ‘create space for spirituality’ and ‘enable an atmosphere of humanity and security around the patient’. Consequently, they were not interpreted as being focused on the personal qualities of the nursing staff.

#### 4.4.5 A Proposed Definition of the Concept of Spiritual Care Based on the Nursing Staff's Description of Spiritual Care in the 2023 Study Compared to the 2003 Definition

In order to visualise the results of the 2023 study in a similar way as the 2003 study, the following definition of the concept of ‘spiritual care’ in a nursing context based on the four themes that emerged during the content analysis, is proposed:

‘Spiritual care’ entails, with suitable nursing interventions, enabling the patient to live out their spirituality, practice their religion, and openly discuss existential questions. Such nursing interventions are characterised by the nursing staff's effort to relate to the whole person and to establish an atmosphere of humanity and safety around the patient. Furthermore, ‘spiritual care’ includes an awareness of the importance of the nursing staff's approach to the implementation of such nursing interventions.

The first part of the definition resembles the first part of the 2003 definition. This part is motivated by the fact that in addition to the concept of ‘spiritual’, the concepts of ‘religion’ and ‘existential questions’ also repeatedly appeared in the respondents' answers and correlated with one of the themes identified in the questionnaire material. As in the 2003 definition, the second sentence of the 2023 definition constitutes a specification of how ‘suitable nursing interventions’ can be understood. This part of the definition is based on the themes identified during the content analysis of the 2023 questionnaire. Broadly speaking, the definitions from the 2003 and 2023 studies are similar to each other, but we do find some substantive differences: the 2023 definition explicitly mentioned that spiritual care is characterised by an effort on the nursing staff's behalf to relate to the whole person (i.e., their patient), and the importance of the nurse's individual approach to spiritual care is emphasised in the 2023 study.

## 5 Discussion

### 5.1 On the Results

Although there are substantial similarities between the results of the 2003 and 2023 studies, as reflected in the respective definitions of the concept of ‘spiritual care’ that were constructed from the content analyses in the two studies, some differences between the two studies remain. In the 2023 study, the importance of the nurse's individual approach to spiritual care was emphasised more than that in the 2003 study. In addition, spiritual care was more clearly characterised as an effort to relate to the whole person than was the case in the 2003 study. It appears that the respondents' understanding of what can or should be included in the framework of spiritual care was broader in the 2023 study compared to that in the 2003 study. Furthermore, there was an increased awareness in the 2023 study that nursing interventions can differ depending on who the patient is and who the caregiver is. The findings of the 2023 study, including that spiritual care relates to the whole person, align, with one exception, with the results of a recently published analysis on 18 international original research articles (published 2010 or later) on how spiritual care is understood within palliative care (Lundberg et al. 2024). The exception is the emphasis on the importance of the nurse's individual approach to spiritual care, which is not found in Lundberg et al.'s results. However, it does align with the increased individualisation of spirituality, accounted for in the introduction. A possible interpretation of this observation is that it reflects a trend in the Swedish society in the direction of an ever‐increasing individualisation and privatisation of spirituality that includes an increasing diversity of expression in people's spirituality (Thurfjell 2015).

In the 2003 and 2023 studies, we noted a distinct difference not only in terms of religious belief (measured by a reported belief in God) but also in terms of how references to ‘organised religiousness’ and ‘non‐organised religiousness’ were distributed between the studies' respondents. This change broadly reflects the social change that took place in Sweden during the same period, according to the WVS. However, the proportion of the nursing staff who reported that they believed in God and regularly attended religious meetings was higher in the oncology clinic than the national average, both in 2003 and 2023. A reasonable interpretation of this observation is that the nursing staff was more religious than the national average in both studies (as measured in term of a reported belief in God). This is important to keep in mind when we compare the definition of ‘spiritual care’ in the 2023 and 2003 studies. The similarities may at least be partially explained by the percentage of nursing staff who reported that their religiousness was relatively high in both studies.

Why the results of the 2023 study (in contrast to those of the 2003 study) emphasised the importance of the nursing staff's efforts in relating to the whole person, as well as the importance of the nurse's individual approach to spiritual care, is of course difficult to say anything with certainty about, but one possible explanation could be that staff whose spirituality is expressed in terms of religiousness may have a narrower understanding of what spiritual care can be, whereas staff who do not subscribe to a traditional (religious) understanding of spirituality, (i.e., spirituality that is expressed without some form of religious practice) may possess a broader understanding of the concept of ‘spirituality’. As there was a statistically significant decrease in religiousness (measured in terms of a reported belief in God) between the 2003 and 2023 studies, this could be expected to be mirrored in the change in understanding of what spirituality is. This explanation is supported by the results of the part of the 2023 study that dealt with attitudes towards spiritual care, where a somewhat unexpected finding from the 2003 study was a significant negative correlation between, on the one hand, the extent to which nursing staff at the respondents' own ward are considered to perform spiritual care and, on the other hand, belief in God. The more the nursing staff reported that they believed in God, the less they perceived spiritual care as being performed in their respective wards (by colleagues) (Lundmark [Link], 2006). This pattern was also observed among those who reported a belief in life after death and those who scored high in ‘non‐organised religiousness’. These correlations suggest that these respondents had a narrower understanding of what spiritual care is than the rest of the staff. These negative correlations were not found in the 2023 study, which supports the interpretation of the findings from 2003 because the proportion of staff who reported a belief in God or life after death decreased substantially between the 2003 study and the 2023 study (Lundmark Forthcoming a).

Since 2003, the awareness of spiritual care might have grown because of the increased research on the topic: the progress of palliative care, which typically includes a holistic care perspective (which in turn typically includes the notion of spiritual care), and changes in the education of healthcare staff. The latter is evident in the Swedish context with the increased presence of texts on spirituality and health in modern textbooks on nursing (see, e.g., Lundmark 2019). However, the picture is mixed. In recent nursing literature that addresses palliative care, the trend seems to be to replace the concept of ‘spirituality’ with ‘existential’ (e.g., Benkel, Molander, and Wijk 2024). How the trends in education might affect the understanding of spiritual care over time is still an uncharted area of research (see also Lundmark Forthcoming b).

In the 2003 study, the participants were provided with a definition of ‘spiritual care’ based on Strang, Strang, and Ternestedt (2002). That study examined how nursing staff at six different healthcare units characterised the meaning of ‘spiritual needs’ and what importance the nursing staff attributed to the spiritual dimension in their work (Strang, Strang, and Ternestedt 2002). In the 2003 study, the topic of spiritual care was examined in an ‘existentially profound’ nursing context. It was argued that, in such a context, the nursing staff would be more sensitive to and find it less challenging to discuss issues of spiritual care. The themes resulting from the content analysis of the respondents' answers were then used to refine the original definition of the concept of ‘spiritual care’ (Lundmark 2003, 2005). The results of the replication study that was conducted in 2023 revealed minor differences that can be explained, at least in part, as a response to the change in the spiritual climate that took place in Sweden between 2003 and 2023 and is reflected in changes in the spirituality of the nursing staff at the oncology clinic. This suggests that definitions that are anchored in a nursing context can change over time for many reasons but may include societal changes (such as an increased sense of individualisation, privatisation of spirituality and a general decline in religiousness in the population). It is important to be aware that definitions of ‘spiritual care’ can be temporally bound, so we can avoid running the risk of assuming that we are talking about the same thing when we talk about spiritual care at different times because that may not be the case.

### 5.2 On Methods, Limitations, Strengths, Generalizability and Future Research

The low response rate in 2023 on the inpatient ward may be attributed to the fact that the participants filled in the questionnaire at work and that the workload in the inpatient ward was higher than that in the outpatient ward at the time of the study (according to the operation managers). However, it is also possible that the different response rates hid a potential bias because we do not know whether those staff members who filled in the questionnaire held the strongest opinions about spiritual care (either strongly for or against spiritual care).

In Section 3.2, concerns were raised that presenting a definition of ‘spiritual care’ in the questionnaire could influence the participants' responses, resulting in reports of opinions that do not actually exist in a Swedish holistic healthcare context. One argument against this concern is that the definition provided in the questionnaire was based entirely on a previous Swedish study (i.e., Strang, Strang, and Ternestedt 2002) of how the nursing staff characterised the spiritual needs of their patients (Lundmark 2005). Although there is no guarantee that the respondents' understanding of spiritual care was not influenced by the definition of ‘spiritual care’ that was provided to them, the rich and diverse answers to the questions that the respondents provided indicated that the respondents could still formulate their own understanding of the concept. Even if the definition provided in the questionnaire did influence the respondents' answers regarding spiritual care, the strength of the replication study lies in the fact that the data can still be compared with the data from the original study because the same questionnaire was used (with minor modifications, accounted for).

As noted in Section 3.2, at the time of the 2003 study, the distinction between religiousness and spirituality was not as distinct as it is today. This becomes evident in how the background information on the participants own spirituality was assessed in the 2003 study where Lundmark primarily addressed what Schnell (2012) labels ‘spirituality with religion’, leaving out what she calls ‘spirituality without religion’. Admittable, if the 2023 study only focused on how nursing staff understand spiritual care in 2023, it would add valuable information to include background questions in the questionnaire that also assessed spirituality without religion. However, since the focus also is on changes in the understandings of spiritual care over time, the importance of being as faithful as possible to the original study design was deemed as primal, perhaps at the price of a shortage on background information concerning the informant's eventual spirituality without religion. Still, the relevant information to compare the studied oncology clinic at 2003 and 2023 with the relevant waves of WVS is there since WVS, when assessing secular values, asked for what, with Schnell's terminology would be labelled ‘spirituality with religion’.

The proposed 2023 definition of the concept of ‘spiritual care’ was based on the understanding of the concept at a Swedish oncology clinic in 2023. Given this background and one of the major results of this study, that is, understandings of ‘spiritual care’ are not entirely stable over time and seem to be sensitive to societal changes, the 2023 definition should be regarded as a snapshot taken at one place, at one time. Have we learnt something that may be valid in other contexts than the studied Swedish oncology clinic? Given the nature of this study's longitudinal design following one particular case (the nursing staff at one specific oncology clinic), the question of generalizability can be reformulated as a question concerning the study's transferability, that is, can the reader transfer knowledge generated from one case to another similar case? (Mertens 2005). A criterion for validity in qualitative research is to ensure that the study has sufficient breadth and depth to generate additional insights into the issue being researched (Yardley 2015). In the present study, the depth of the study was ensured by providing sufficient background information about the clinic, some relevant information from the WVS about Swedish society, information about the study's theoretical framework and method and a detailed account of the results of the study. The reader has thus been provided with sufficient information to draw their own comparisons with other cases (Mertens 2005). In this context, two issues in particular should be considered. First, it is important to consider Sweden's noticeably extreme position in the WVS. The extent to which the results of the presented study can be transferred to other cases in other countries should be informed by the similarities and differences between Sweden and the other countries in question. Second, it is important to consider the significant presence of palliative elements at oncology clinics. In the 2003 study, the decision to perform a study at an oncology clinic was motivated by the extensive palliative elements present at the clinic and the fact that the patients' average stay at the clinic was quite long. These circumstances allowed the patients and their caregivers to get to know each other well. The level of personal confidence between the clinic's staff and the patients was considered relatively high. Furthermore, it was argued that the general uncertainty about their future or certainty about their impending death that many of the clinic's patients experienced would create conditions conducive to spiritual and existential reflections. Such reflections could also be expected to be expressed in conversations between the nursing staff and their patients (Lundmark 2006). Given these considerations, Lundmark presented two arguments for the possibility of generalisation of the study's results. First, the claim to generalisability rested on a basic methodological assumption borrowed from William James' research in the psychology of religion called ‘the method of the extreme cases’, according to which a few extreme cases can provide good general knowledge about most non‐extreme cases (Hood, Hill, and Spilka 2018). Second, the situation at the oncology clinic could be regarded as extreme in the sense that the palliative elements and interventions used in that environment were profound. Reflections about spiritual care that were expressed by the nursing staff may, therefore, be considered relatively well founded. If this assumption was correct, Lundmark argued, the results of the study probably gave insight into a broader nursing context than the context in which the study was performed (Lundmark 2005, 2006). Following this line of argument, one would expect the results of this study to reflect a more general view of the phenomenon, but perhaps in a more condensed form (see also Mascio et al. 2024, for a similar discussion on the method of extreme cases in the context of studying spiritual care). As a methodological question, this deserves more research because many factors may co‐vary with each other in such a research context. For example, on the one hand, the decrease in religiousness among the nursing staff between 2003 and 2023 might be the cause for the small but noticeable changes in their understanding of spiritual care at the oncology clinic, but on the other hand, it might be the case that people who perceive themselves as spiritual (or religious) are more interested in working with palliative care than people who do not perceive themselves as spiritual. In the latter case, the existentially loaded situation that exists in palliative care might not be what triggers the nurses' reflections on spirituality and healthcare but is simply a result of an overrepresentation of staff who are interested in these questions.

Another issue that can further complicate the scenario outlined above, especially given that Sweden (and the rest of the Nordic region) stands out in many ways in WVS's measurements compared to most other countries, is the observation that the culture in which a definition of ‘spiritual care’ is constructed can also be influential. This last point is a question for future research and may be interrogated by the question: What would a definition of the concept of ‘spiritual care’ look like if it were constructed using a method similar to the method used in the present study but where the spiritual climate was radically different?.

To the best of my knowledge, this is the first longitudinal study ever done addressing questions on changes in understanding of spiritual care over time. Will similar changes in understandings of spiritual care over time occur if similar studies would be conducted in less secularised areas of the world?

## 6 Conclusions

Concerning RQ1, the content analysis revealed four themes and several sub‐themes. The four themes were (i) ‘relate to the whole person’, (ii) ‘enable an atmosphere of humanity and security around the patient’, (iii) ‘enable the patient to live out their spirituality and practice their religion’ and (iv) ‘be aware of the importance of one's own approach to spiritual care’. These themes were incorporated in a proposed definition of ‘spiritual care’, valid at least for the studied oncology clinic at the time it was assessed:

‘Spiritual care’ entails, with suitable nursing interventions, enabling the patient to live out their spirituality, practice their religion, and openly discuss existential questions. Such nursing interventions are characterised by the nursing staff's effort to relate to the whole person and to establish an atmosphere of humanity and safety around the patient. Furthermore, ‘spiritual care’ includes an awareness of the importance of the nursing staff's approach to the implementation of such nursing interventions.

Concerning RQ2, the nursing staff's understanding of the concept of ‘spiritual care’ in the 2003 and 2023 studies revealed several similarities but also a number of small but noticeable differences. The importance of the nurses' individual approach to providing spiritual care was emphasised more in the 2023 study, including an increased awareness that nursing interventions can be different depending on who the patient is and who the caregiver is. Furthermore, spiritual care was more clearly characterised by an effort to relate to the whole person in 2023. Finally, the nurses' understanding of what can or should be included in a framework of spiritual care seems to be broader in 2023.

The answers this paper provides for RQ1 and RQ2 indicate that the concept of ‘spiritual care’ is not entirely stable over time and is sensitive to societal changes, such as secularisation. This means that, in addition to considering cultural and other contextual differences when one studies understandings of the concept of ‘spiritual care’ in a nursing context or when one evaluates other research studies on the topic, the lack of stability of the concept of ‘spiritual care’ over time should be taken into account. This demands that one be sensitive to the historical context of the study and be cognizant of any societal changes that may have taken place.

A final conclusion that can be drawn from this study is that because the understanding of spiritual care has changed in the presented case, it can very well have done so also in other cases.

## Conflicts of Interest

The author declares no conflicts of interest.

## Data Availability

The data that support the findings of this study are available from the corresponding author upon reasonable request.
